# One Year With *WAO *Journal

**DOI:** 10.1097/WOX.0b013e318194c119

**Published:** 2008-12-15

**Authors:** Johannes Ring, Lanny Rosenwasser

**Affiliations:** 1 Editor in Chief; 2 Co-editor in Chief

## 

**Johannes Ring:**
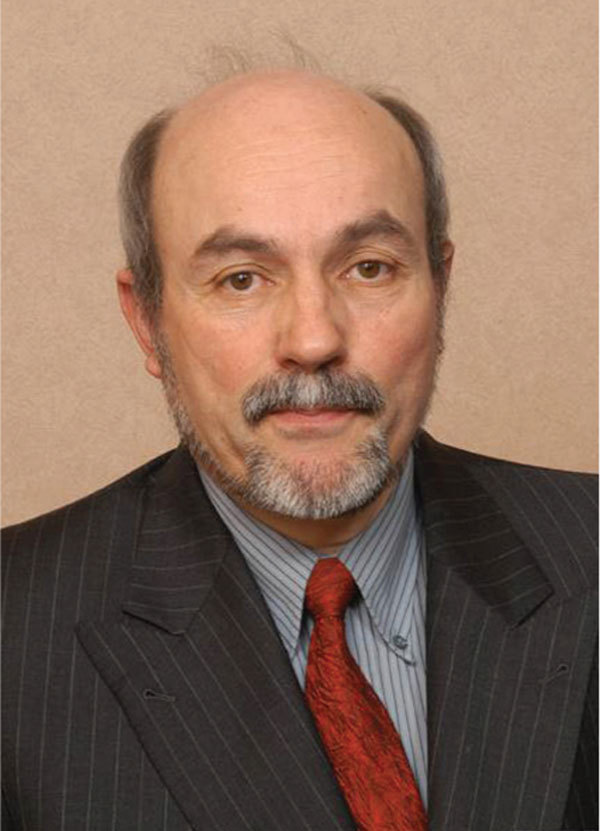
*Johannes Ring (Editor in Chief)*

**Lanny Rosenwasser:**
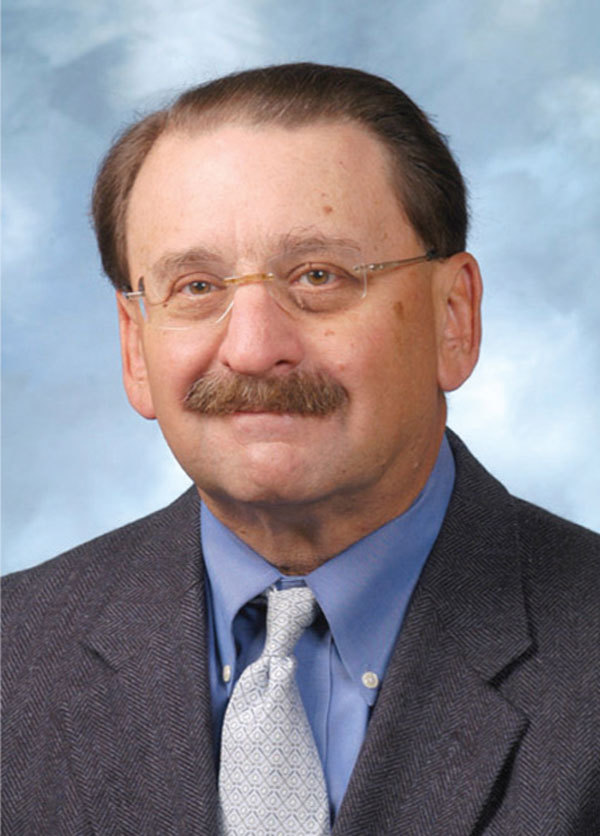
*Lanny Rosenwasser (Co-editor in Chief)*

One year ago at the World Allergy Congress in Bangkok, the new *Journal of the World Allergy Organization *(WAO) was officially launched; the first issue of *WAO Journal *appeared in January 2008. In several aspects, this was a major move and a great decision for *WAO*: After 20 years with *Allergy Clinical Immunology International *(first *Allergy Clinical Immunology News*)--*Journal of World Allergy Organization *(*ACII-JWAO*), the board of directors decided to open totally new doors and launch a new electronic-only journal. We can reach 30,000 practicing allergists worldwide via e-mail, allowing a much higher degree of flexibility and actuality. On the other hand, many of our readers still like paper and have to get used to the new electronic style. We believe, however, that this is the future of scientific communication and interchange! Both for *WAO *and for the publisher, it is an experiment; there are still just few electronic-only journals as official organs of national or international specialty societies. However, we are confident that *WAO *is on the road to success.

With approximately 40 articles published in this first year--most of them of highest quality--*WAO *covered large parts of the broad spectrum of the interdisciplinary field of allergy, both in research and clinics.

*WAO *is in progress; under the new president, Walter Canonica--in the succession of Mike Kaliner, Carlos Baena Cagnani, Allen Kaplan, Gunnar Johansson, and so many famous allergists--the organization has become the leading force in international allergology, achieving much more than organizing a large congress every other year. *WAO *is the platform for everybody who wants to contribute to advance excellence in patient care, research, and education in the field of allergy and clinical immunology.

Not only *WAO *is in progress, but also allergy is rapidly developing.

The bad news first. There is still an increasing prevalence of many allergic diseases all over the world, also in countries where these diseases were rather rare 20 years ago. At the same time, allergic diseases become more and more complex and difficult to diagnose and treat; furthermore, there seems to be an age shift with not only children and young adults affected but more and more adults and elderly persons suffering from severe allergies.

Allergen-specific immunotherapy (ASIT) has considerably improved in quality and efficacy and safety; however, it is applicable only to a small number of allergic patients. So many people are suffering from allergies, where ASIT does not exist or is not available.

There are also good news. As rapidly as allergy prevalence was growing, the progress in allergy research is going on--we just want to mention the discovery of gene loci closely associated with allergic diseases as the cytokine cluster on chromosome 5, the ADAM 33 for bronchial hyperreactivity or the filaggrin mutations for atopic eczema. Progress has been made in ASIT with new extracts, adjuvants, and route of application.

There is also great progress in education, not only for medical students and doctors but also for patients and patient organizations. Educational programs have been developed successfully--some of them with proven efficacy in randomized prospective trials--for asthma and eczema and are being started for anaphylaxis. On the basis of this improved education of doctors, better clinical care for allergic individuals has been achieved in many countries of the world; yet, there is still a lot to do!

*WAO Journal *considers itself to be *one *tool in the big effort to improve the lives of allergic patients all over the world.

At the end of this year, it is time to thank so many people involved in this undertaking, first of all, not only the *WAO *board of directors and the staff in Milwaukee (with Charu Malik, Lorie Conwell, and the editorial office) but also the people from the publisher (Diana Pesek, Adam Nicely), the regional associate editors, and the members of the editorial board who have helped to shape *WAO*, and last but not least the authors who have submitted excellent papers and the reviewers who have helped to make them even better!

With all our best wishes for the coming holidays and a happy, healthy, and peaceful New Year 2009!

Johannes Ring

(Editor in Chief)

Lanny Rosenwasser

(Co-editor in Chief)

